# Vitamin D Deficiency Is Prevalent in Morbidly Obese Adolescents Prior to Bariatric Surgery

**DOI:** 10.1155/2013/284516

**Published:** 2013-02-24

**Authors:** Marisa Censani, Emily M. Stein, Elizabeth Shane, Sharon E. Oberfield, Donald J. McMahon, Shulamit Lerner, Ilene Fennoy

**Affiliations:** ^1^Department of Pediatrics, Columbia University Medical Center, 622 West 168th Street, PH 5E-522, New York, NY 10032, USA; ^2^Department of Medicine, Columbia University Medical Center, New York, NY 10032, USA

## Abstract

*Background*. Obese adults are frequently vitamin D deficient before bariatric surgery; whether similar abnormalities exist in morbidly obese adolescents is unknown. *Objective*. To determine the prevalence of vitamin D deficiency in morbidly obese adolescents. *Methods*. Cross-sectional study of preoperative laboratory measures from 236 adolescents evaluated for bariatric surgery. *Results*. The group (*N* = 219 with 25-hydroxyvitamin D (25OHD) and parathyroid hormone (PTH) levels; 76 boys, 143 girls; 15.9 ± 1.2 years; 43% Caucasian, 35% Hispanic, and 15% African American) had mean BMI of 47.6 ± 8.1 kg/m^2^. 25OHD levels were deficient (<20 ng/mL) in 53%; 8% had severe deficiency (<10 ng/mL); only 18% of patients were replete (>30 ng/mL). 25OHD levels were inversely associated with BMI (*r* = −0.28, *P* < 0.0001) and PTH levels (*r* = −0.24, *P* = 0.0003). Race was the strongest predictor of 25OHD (*P* < 0.002); 82% of African Americans, 59% of Hispanics, and 37% of Caucasians were deficient. African American race, BMI, and PTH explained 21% of the variance in 25OHD (*P* < 0.0001). *Conclusions*. Most adolescents presenting for bariatric surgery have suboptimal vitamin D levels, with African Americans and those with higher BMIs at greatest risk for vitamin D deficiency. All morbidly obese adolescents should be screened for vitamin D deficiency before bariatric procedures.

## 1. Introduction

Over the past thirty years, the adolescent obesity rate has more than tripled. It has been estimated that 17% of US children and adolescents meet criteria for overweight (body mass index (BMI) between the 85th–95th percentile for age) and 4% are now considered morbidly obese (BMI > 99th percentile) [1, 2]. Bariatric surgery is widely used in the morbidly obese adult population [3–5]. Since the late 1990s, coincident with the increased prevalence of obesity (BMI > 30 kg/m^2^) in the adolescent population, bariatric surgery use in adolescents has grown rapidly. It has been estimated that between 1000 and several thousand adolescents undergo bariatric procedures each year [6]. There is increasing evidence suggesting that these procedures may be the most effective treatment for weight loss in the adolescent as well as in the adult [7–12]. Indeed, a recent meta-analysis found that in adolescents, bariatric surgery was associated with permanent weight loss and resolution of concomitant metabolic conditions, including diabetes and hypertension [11]. 

Data on skeletal and mineral metabolism consequences of morbid obesity and bariatric surgery in adolescents are limited. Prior to surgery, obese adults are often vitamin D deficient, with lowest levels in the most obese individuals [13]. Inadequate calcium intake and secondary hyperparathyroidism are common [13–16]. The only data available in morbidly obese adolescents (BMI > 35 kg/m^2^) comes from a small study that did not describe the frequency of vitamin D deficiency, although mean vitamin D levels were in the insufficient range [17]. Vitamin D deficiency may have important consequences in the adolescent, as vitamin D plays an essential role in calcium absorption from the small intestine and in the development and maintenance of the skeleton. It has been associated with rickets, or the failure of mineralization of developing bone and cartilage, in growing children and with osteomalacia in adults [18]. Nonskeletal associations of vitamin D deficiency have also been documented, with low serum 25-hydroxyvitamin D (25OHD) levels correlating directly with the degree of insulin resistance, hypertension, and progression to diabetes mellitus [19, 20]. 

 There are no guidelines in adult or adolescent populations undergoing bariatric surgery for the diagnosis or prevention of skeletal consequences of bariatric procedures. Further, the prevalence of mineral metabolism abnormalities in adolescents presenting for bariatric surgery is not known. The primary aim of this study was to determine the prevalence of vitamin D deficiency in morbidly obese adolescents who present for bariatric surgery. Secondarily, we sought to identify patient characteristics that might represent predictors of hypovitaminosis D in this population.

## 2. Materials and Methods

All 236 pediatric patients evaluated for restrictive bariatric surgery at our institution between March 2006 and June 2011 were included. Patients without 25OHD and PTH levels available (*n* = 17) were excluded from the study. Patients were referred from the weight management programs at Columbia University Medical Center or by private pediatricians. Eligible subjects were adolescents between the ages of 14 and 18 years who had BMI > 40 kg/m^2^ or > 35 kg/m^2^ and at least one comorbidity. Since children of the same sex and chronological age may differ by skeletal age and pubertal stage, only patients with tanner stage IV-V of puberty and documented skeletal maturity with a bone age of at least 13.5 years in females and 14.5 years in males were eligible for bariatric surgery. Other eligibility criteria included: a history of obesity for at least five years with documented failed attempts at diet and lifestyle management, emotional maturity, and willingness to comply with the program's protocols. All patients signed informed consent under a Columbia University Medical Center Institutional Review Board approved protocol. 

At the first clinic visit, demographic data, detailed medical history, physical exam, and laboratory studies including serum calcium, phosphorus, alkaline phosphatase, 25OHD, 1,25 dihydroxyvitamin D (1,25(OH)_2_D), and parathyroid hormone (PTH) were obtained and entered into a database. 

### 2.1. Analytical Methods

Laboratory studies were performed at Esoterix Laboratory (Calabasas Hills, CA). Serum 25OHD was measured by liquid chromatography-tandem mass spectrometry with an interassay coefficient of variation (CV) of <10%. The lower limit of quantitation of the method is 1 ng/mL. Serum 1,25(OH)_2_D was measured by radioreceptor assay (CV 5.6–17.0%). PTH was measured by a 2-site immunochemiluminometric method (CV 9.9–19.0%). Serum calcium was measured using spectrocolorimetric method (CV < 10%). 

### 2.2. Statistical Analysis

Analyses were conducted with SAS version 9.1 (SAS institute Inc., Cary, NC). Two-sided *P* values < 0.05 were considered to indicate statistical significance. Data are presented as mean +/−SD except where otherwise noted. Spearman's correlation and linear regression were used to assess the relationship between vitamin D levels and different bone parameters. Analysis of variance and Chi-square test were used to compare groups of patients by demographic characteristics. Variables found to be significantly associated with 25OHD in univariate analysis (BMI, African American race, PTH; *P* < 0.05) were entered into a stepwise regression model. For the analysis of change in serum 25OHD level by season, subjects were grouped according to race (African American versus non-African American) for winter (December through February), spring (March through May), summer (June through August), and autumn (September through November). In this study, vitamin D deficiency was defined as a serum 25OHD level <20 ng/mL, severe vitamin D deficiency <10 ng/mL, and vitamin D insufficiency as a level <30 ng/mL but ≥20 ng/mL [20]. 

## 3. Results

**Figure 1:**
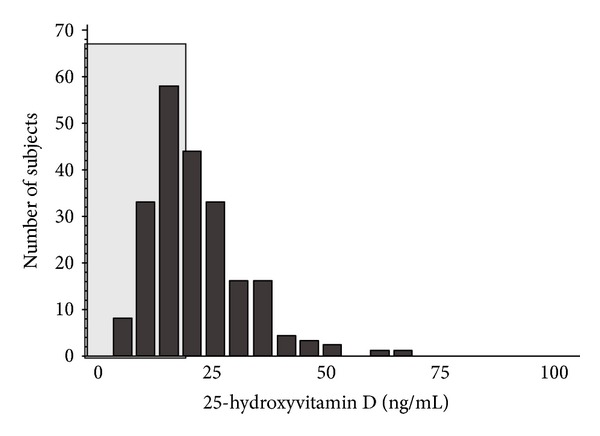
The distribution of 25-hydroxyvitamin D (25OHD) serum concentrations in 219 morbidly obese adolescents; shaded area indicates 25OHD level below 20 ng/mL.

Of the 236 patients at baseline, 219 patients had 25OHD and PTH levels available for review and constituted our cohort. The excluded patients did not differ from the included patients in mean age, mean body mass index (BMI), sex, or race. The clinical characteristics of the cohort are summarized in Table 1. Patients were not using supplements at the time of the study visit. Mean serum 25OHD was 20.7 ± 9.8 ng/mL, and more than half of all subjects (53%) were vitamin D deficient (<20 ng/mL; Figure 1). Severe deficiency (25OHD < 10 ng/mL) was found in 8%, while only 18% of subjects had 25OHD levels >30 ng/mL. Vitamin D deficiency tended to be more common in boys (62% of males versus 48% of females; *P* = 0.07) but did not reach statistical significance. 

**Figure 2:**
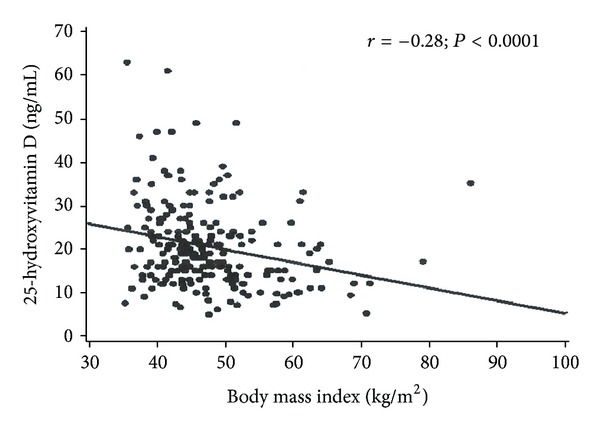
The association (Spearman correlation coefficient (*r*)) between 25-hydroxyvitamin D (25OHD) concentration and body mass index (BMI).

**Figure 3:**
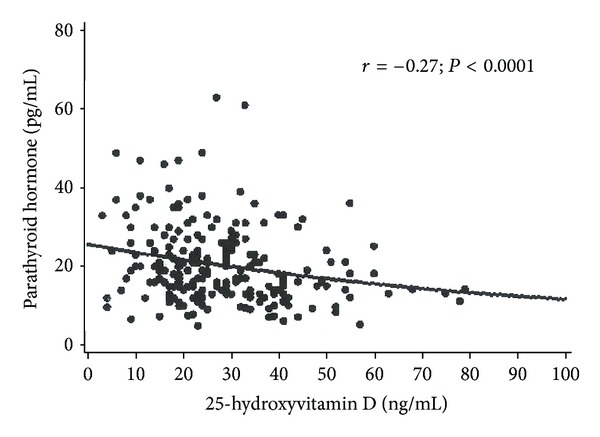
The association (Spearman correlation coefficient (*r*)) between parathyroid hormone (PTH) levels and 25-hydroxyvitamin D (25OHD) concentration.

Greater BMI was associated with lower serum 25OHD (*r* = −0.28, *P* < 0.0001; Figure 2). This relationship persisted after controlling for PTH (*r* = −0.22, *P* < 0.002). The expected inverse association between PTH and 25OHD was observed (*r* = −0.24, *P* = 0.0003; Figure 3). Despite the generally low levels of serum 25OHD, only 8 patients (3.7%) had clear evidence of secondary hyperparathyroidism (PTH > 55 pg/mL). PTH levels were directly associated with BMI (*r* = 0.23, *P* = 0.0006), a relationship that persisted after controlling for 25OHD (*r* = 0.18, *P* < 0.01).

**Figure 4:**
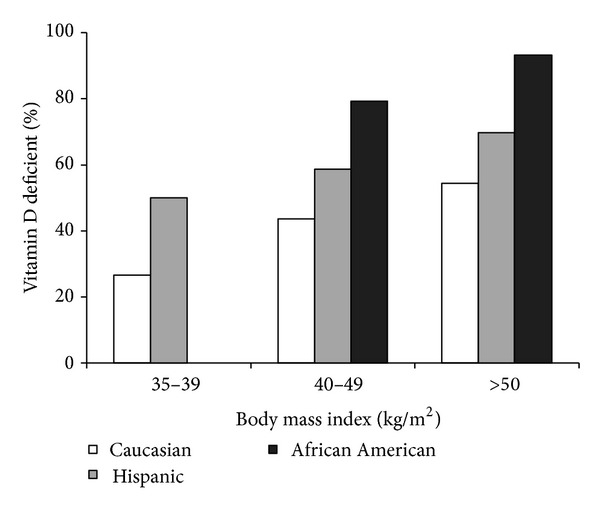
Prevalence of 25-hydroxyvitamin D deficiency (25OHD < 20 ng/mL) according to race and body mass index (BMI).

Race was also associated with vitamin D deficiency (*P* < 0.002) with 25OHD concentrations <20 ng/mL particularly common among African American patients (81% of African Americans, 59% of Hispanics, and 37% of Caucasians; *P* < 0.001). All African American subjects had 25OHD levels <30 ng/mL, as compared to 84% of Hispanic patients and 75% of Caucasian patients (Figure 4). African American race was independently associated with decreased 25OHD levels (*r* = −0.28, *P* < 0.0001). Of note, there were no African American patients with a BMI < 40 kg/m^2^. However, racial differences persisted after controlling for BMI.

There was seasonal variability in 25OHD levels in the cohort as a whole (Table 2; ANOVA *P* < 0.01), with lower 25OHD levels in winter than in summer (*P* < 0.03). Compared with other ethnicities, African Americans were found to have 25OHD levels in the deficient range in all seasons. Mean serum 25OHD levels were significantly lower in African American subjects compared with non African Americans both when unadjusted (*P* = 0.0003) and adjusted for season and the race/season interaction (*P* = 0.0007).

Significant predictors of vitamin D deficiency in the univariate analysis included BMI, African American race, and PTH. These variables were entered into a stepwise linear regression model and were found to explain 21% of the overall variance in 25OHD (overall model: *P* < 0.0001). After multivariable adjustment, 25OHD deficiency was significantly associated with BMI (*β* coefficient (standard error (SE)): −0.33 (0.12), *P* < 0.006) and race (−6.26 (1.96), *P* < 0.002), with race found to be the strongest predictor of 25OHD. PTH did not reach statistical significance in the regression model (−0.1 (0.06), *P* < 0.08). Each increase in BMI of 1 kg/m^2^ was associated with a decrease of 0.35 ng/mL in 25OHD. Age, sex, serum calcium, and 1,25(OH)_2_D were not found to be significant predictors of 25OHD. 

## 4. Discussion

In this cohort of over 200 morbidly obese adolescents presenting for bariatric surgery, over half of all subjects were frankly vitamin D deficient, and only 20% were clearly vitamin D replete. These data extend to the adolescent population that previously reported findings in morbidly obese adults, where a high prevalence of vitamin D deficiency was documented, and the extent of deficiency was predicted by degree of obesity and race, with those having the highest BMI and of African American race most likely to be deficient [13]. Identification of vitamin deficiencies is particularly important in the bariatric population, as these patients may be at greater risk of postoperative vitamin deficiencies. Weight loss has been associated with bone loss, and malabsorption may occur with certain bariatric procedures further contributing to deficiency risk. 

To our knowledge, this study is the first to assess the prevalence of vitamin D deficiency in a cohort of morbidly obese (BMI > 35 kg/m^2^) adolescent bariatric surgery candidates in relation to ethnicity, degree of obesity, sex, seasonal variability, and PTH levels. Although the prevalence of low vitamin D status among normal weight, overweight, and obese adolescents has been addressed in previous studies, [21–23] the effect of increasing obesity level on vitamin D status in extreme obesity and the relationship between vitamin D and PTH in this population has not been described in detail. In one study of a population of morbidly obese adolescents undergoing laparoscopic gastric banding, vitamin D levels were reported in preoperative adolescents (*n* = 45), with mean baseline levels slightly higher than those reported here [17]. Serum 25OHD rose one year after laparoscopic adjustable gastric banding (from 22 ± 10 pg/mL to 26 ± 12 pg/mL, *P* = 0.025) with no change in PTH levels one year after laparoscopic adjustable gastric banding. However, the study did not report incidence of insufficient or deficient 25OHD levels, baseline PTH levels, data on postoperative vitamin D supplementation, or patient characteristics that were associated with hypovitaminosis D in this at risk population. 

The BMI guidelines used for determining appropriate adolescent patients to screen for bariatric surgery were consistent with NIH criteria for bariatric surgery in adults [24]. In this context, we had the ability to stratify our population into three different categories of obesity (35–39 kg/m^2^, 40–49 kg/m^2^, and >50 mg/m^2^) and analyze ethnic groups within each BMI cohort. BMI was found to be a strong determinant of serum 25OHD concentrations, with higher BMI associated with lower 25OHD. In our cohort (mean BMI range 35.3–86.2 ± 8.1), an increase in BMI of 1 kg/m^2^ was associated with a decrease of 0.35 ng/mL in 25OHD. Potential explanations for this association have been proposed, including decreased sunlight exposure and sedentary lifestyle, inadequate dietary intake, and decreased bioavailability of vitamin D secondary to sequestration of fat soluble vitamin D in excess adipose tissue [18, 25–29]. 

Our finding that vitamin D deficiency was most common in African Americans is in accordance with previous literature addressing racial differences in vitamin D metabolism in adolescents and adults of varying adiposity [29–32]. The lower serum 25OHD in African American patients has been attributed to increased melanin pigment interfering with the absorption of ultraviolet B light. This leads to diminished dermal production of vitamin D and subsequent decreased hepatic synthesis of the major circulating metabolite 25OHD.

There is no consensus concerning the definition of vitamin D deficiency in adults, and data in pediatric literature are sparse [18]. However, studies have reported impaired calcium absorption and lower bone mineral density at 25OHD levels of <32 ng/mL [33–36]. Holick has defined vitamin D deficiency as 25OHD <20 ng/mL and vitamin D insufficiency as 25OHD of 21 to 29 ng/mL in adults [20]. In adults, serum PTH concentrations rise as 25OHD concentrations fall below 31–32 ng/mL [36, 37]. In an adult population presenting prior to bariatric surgery, Stein et al. reported that the association between PTH and BMI was mediated by 25OHD, suggesting that the hyperparathyroidism seen in extremely obese adults was largely secondary to vitaminD insufficiency [13]. El-Hajj Fuleihan et al. studied healthy children in Lebanon and showed similar findings to the adult literature and recommended that 25OHD levels are >20–30 ng/mL to maintain serum PTH below the upper limit of normal and avoid complications of secondary hyperparathyroidism [38]. However, the relationship between PTH and 25OHD is not as clear in the pediatric population as in adults. Pediatric studies have been unable to clearly identify an inflection point of serum 25OHD for maximal suppression of serum PTH [39]. In a study of 51 obese African American female adolescents, there was no association between 25OHD and PTH [40]. 

Although a significant inverse association between PTH and 25OHD was seen in our morbidly obese adolescent population, only a small percentage of patients (3.7%) had clear evidence of secondary hyperparathyroidism. Our study confirms findings by Gordon et al. who documented an inverse relationship between 25OHD and PTH levels in 307 normal weight adolescents without increased PTH concentrations [30]. The clinical significance of low 25OHD concentration without concomitant secondary hyperparathyroidism is still largely unknown in children and adolescents. Although the explanation for this finding is unclear, the adolescent vitamin D deficiency detected may be a more acute finding as opposed to long standing vitamin D deficiency in adults with chronic PTH elevation. This finding may also reflect the fact that the laboratory normal range for PTH is likely to significantly overestimate normal levels for children and adolescents, as it was calculated from populations that included older individuals with mild renal impairment that is likely to have led to higher PTH levels. In our cohort, only 8 patients (3.7%) had vitamin D levels <30 ng/mL with clear evidence of secondary hyperparathyroidism based on the laboratory normal range (PTH > 55 pg/mL). However, a higher percentage of our patients (16.9%) had PTH levels in the upper third of normal (>40 pg/mL), with 89% of these patients having vitamin D levels <30, lending support to this hypothesis. 

The role of parathyroid hormone in morbid obesity is also unclear. We found that PTH levels remained positively associated with BMI, even after controlling for 25OHD levels. Snijder et al. found similar results and suggested that PTH may contribute to the development of adiposity independently of 25OHD. It has been hypothesized that excess PTH could promote further weight gain by enhancing lipogenesis [26]. 

Our data demonstrate seasonal variability in vitamin D status, with the highest 25OHD concentrations in subjects enrolled in the summer and lowest concentrations in subjects enrolled in the winter regardless of race. Of note, the prevalence of vitamin D deficiency was higher among African American subjects, compared with other subjects, during all seasons: winter (89% versus 70%), spring (89% versus 49%), summer (71% versus 33%), and autumn (75% versus 44%), indicating that African Americans may be at higher risk for low vitamin D status throughout the year. The seasonal variation of 25OHD levels is consistent with previously reported findings in adults and normal weight adolescents and extends these findings to the morbidly obese adolescent population [13, 30, 32]. Although we had no direct measurement of sun UV exposure, the data do support an association between sun exposure and vitamin D levels in morbidly obese adolescents. 

This study has some important limitations, including the lack of a community based nonobese adolescent control group. Another limitation is the study's cross-sectional design which prevents the assessment of the temporal associations identified between vitamin D levels and risk factors for hypovitaminosis D. Further, we do not have data regarding dietary intake of vitamin D and calcium or data on the consequences of abnormal vitamin D status on bone density or quality in these subjects. Despite these limitations, these data provide insight into prevalence and risk factors for vitamin D deficiency in obese adolescents before bariatric surgery. 

## 5. Conclusions

In conclusion, we found a high prevalence of vitamin D deficiency among obese adolescents presenting for bariatric surgery. Individuals who were African American and had higher BMIs were at greatest risk for vitamin D deficiency. There are currently no established guidelines in adult or adolescents undergoing bariatric surgery for key issues pertaining to the detection and prevention metabolic bone disease, such as the monitoring of biochemical indices at baseline and after surgery or when to start supplementation. These results support screening all morbidly obese adolescents for vitamin D deficiency, and repleting those who are deficient. This is particularly important prior to bariatric surgery where weight loss and decreased calcium and vitamin D absorption in some procedures may place these patients at further risk. These results also highlight the need for more data in order to determine an optimal regimen for vitamin D repletion in morbidly obese adolescents prior to bariatric surgery. Procedure-specific information on the postoperative effects on vitamin D in adolescents is also needed.

## Figures and Tables

**Table 1:** Association of patient characteristics (*n* = 219) with 25-hydroxyvitamin D (25OHD) levels.

Variable	Mean ± SD (range)	Correlation with 25 OHD^†^	
		*r*	*P*
Age (years)	15.9 ± 1.2 (12.9–18.5)	− 0.04	0.59
Sex (female : male)	143 : 76	0.12	0.07
**Race (% African American)**	**15%**	**−0.28**	**<0.0001**
**BMI (kg/m** ^ 2^ **)**	**47.5 **±** 8.1 **(35.3–86.2)	**−0.28**	**<0.0001**
Serum calcium^‡^ (8.7–10.2 mg/dL)	9.3 ± 0.6	0.04	0.53
**iPTH (10**–**55 pg/mL)**	**28.0 **±** 13.9**	**−0.24**	**0.0003**
1,25(OH)_2_D (15.0–90.0 pg/mL)	**46.13 **±** 15.2**	** 0.07**	**0.34**

Normal ranges presented in parentheses for serum measurements.

^†^ *r* (Spearman correlation coefficient) and *P* values presented for the association between each variable and total 25OHD concentration in the univariate analysis. Variables with significant correlations are in boldface.

^‡^Calcium values are corrected for serum albumin.

**Table 2:** Serum 25-hydroxyvitamin D levels (ng/mL): distribution by season and race.

Season†	All subjects (*N* = 219)*			African American (*n* = 33)			Non-African American (*n* = 186)**		
	*N*	Avg ± SD	% <20	*N*	Avg ± SD	% <20	*N*	Avg ± SD	% <20
Winter	42	16.9 ± 7.4	74	9	13.7 ± 3.8	89	33	17.8 ± 7.9	70
Spring	70	20.7 ± 9.7	54	9	13.6 ± 5.1	89	61	21.7 ± 9.8	49
Summer	56	22.9 ± 11.4	38	7	17.0 ± 5.0	71	49	23.7 ± 11.8	33
Autumn	51	21.3 ± 9.1	49	8	16.5 ± 6.3	75	43	22.2 ± 9.4	44

ANOVA *P value < 0.01; **P value < 0.02.

^†^Winter (December through February), spring (March through May), summer (June through August), and autumn (September through November).
